# Screening for Prognostic microRNAs Associated with Treatment Failure in Diffuse Large B Cell Lymphoma

**DOI:** 10.3390/cancers14041065

**Published:** 2022-02-20

**Authors:** Leyre Bento, Oliver Vögler, Adriana Sas-Barbeito, Josep Muncunill, Teresa Ros, Jordi Martínez, Adriana Quintero-Duarte, Rafael Ramos, Víctor Jose Asensio, Concepción Fernández-Rodríguez, Antonio Salar, Alfons Navarro, Raquel del Campo, Javier Ibarra, Regina Alemany, Antonio Gutiérrez

**Affiliations:** 1 Department of Hematology, Son Espases University Hospital, 07120 Palma, Spain; leyre.bento@ssib.es (L.B.); maria.ros@ssib.es (T.R.); jorgej.martinez@ssib.es (J.M.); 2 Group of Clinic and Biology of Hematological Neoplasms, Health Research Institute of the Balearic Islands (IdISBa), 07010 Palma, Spain; adrianasas95@gmail.com (A.S.-B.); rcampo@hsll.es (R.d.C.); 3 Group of Advanced Therapies and Biomarkers in Clinical Oncology, Research Institute of Health Sciences (IdISBa-IUNICS), University of the Balearic Islands, 07122 Palma, Spain; oliver.vogler@uib.es; 4 Group of Clinical and Translational Research, Department of Biology, University of the Balearic Islands, 07122 Palma, Spain; 5 Group of Genomics-Bioinformatics Platform, Health Research Institute of the Balearic Islands (IdISBa), 07010 Palma, Spain; josep.muncunill@ssib.es; 6 Department of Pathology, Son Espases University Hospital, 07120 Palma, Spain; amquintero@ssib.es (A.Q.-D.); rafaelf.ramos@ssib.es (R.R.); 7 Molecular Diagnosis and Clinical Genetics Unit (GENIB), Son Espases University Hospital, 07120 Palma, Spain; victor.asensio@ssib.es; 8 Group of Health Genomics, Health Research Institute of the Balearic Islands (IdISBa), 07010 Palma, Spain; 9 Department of Pathology, Hospital del Mar-IMIM, 08003 Barcelona, Spain; mconcepcionfernandezrodriguez@psmar.cat; 10 Group of Applied Clinical Research in Hematology, Cancer Research Program-IMIM, Hospital del Mar Medical Research Institute, 08003 Barcelona, Spain; asalar@psmar.cat; 11 Molecular Oncology and Embryology Laboratory, Human Anatomy Unit, Faculty of Medicine and Health Sciences, University of Barcelona, 08036 Barcelona, Spain; anavarroponz@ub.edu; 12 Department of Hematology, Hospital del Mar-IMIM, 08003 Barcelona, Spain; 13 Department of Hematology, Son Llàtzer University Hospital, 07198 Palma, Spain; 14 Department of Pathology, Son Llàtzer University Hospital, 07198 Palma, Spain; jibarra@hsll.es

**Keywords:** diffuse large B cell lymphoma, prognosis, epigenetic markers, chemoresistance, microRNA

## Abstract

**Simple Summary:**

Around 30–40% of patients with diffuse large B cell lymphoma suffer early relapse after standard chemotherapy, but today no prediction whether a patient belongs to this group is possible. MicroRNA are small nucleotide sequences that regulate cellular functions via post-transcriptional modification of gene expression and can serve as prognostic biomarkers. A novel two-step strategy first used a small patient discovery group to identify possible microRNA candidates by comparing their levels in chemosensitive and chemoresistant patients via microarray. Overexpression of these microRNA was then analyzed in a large patient cohort and, as a result, three new microRNA biomarkers with prognostic potential could be identified. Early identification of those patients being at risk of failure with standard therapy is a prerequisite to develop more efficient treatments and a step towards precision medicine.

**Abstract:**

Diffuse large B cell lymphoma (DLBCL) treatment with R-CHOP regimen produces 5-year progression-free survival and overall survival of around 60–70%. Our objective was to discover prognostic biomarkers allowing early detection of the remaining 30–40% with poor long-term outcome. For this purpose, we applied a novel strategy: from a cohort of DLBCL patients, treated with standard therapy, a discovery group of 12 patients with poor prognosis (advanced stage III–IV, R-IPI > 2) was formed, consisting of six chemoresistant (refractory/early relapse < 12 months) and six chemosensitive (complete remission > 3 years) subjects. By using microarray assays, the most differentially expressed miRNAs were defined as an initial set of prognostic miRNA candidates. Their expression was then analyzed in a validation cohort of 68 patients and the three miRNAs with the most significant impact on event-free and overall survival were selected. In the DLBCL cell line U-2932 the transfection with miR-1244 and miR-193b-5p, but not miR-1231, blocked the effect of CHOP on cell viability. A subsequent gene set enrichment analysis in patients revealed the implication of the first two miRNAs in cell cycle control and chemoresistance-related pathways, whereas the last one was involved in immunological processes. In conclusion, this novel strategy identified three promising prognostic markers for DLBCL patients at high risk of failure with standard therapy.

## 1. Introduction

Diffuse large B cell lymphoma (DLBCL) is the most common subtype of lymphoid neoplasm characterized as a heterogeneous group of aggressive lymphomas. Treatment of DLBCL is relatively homogeneous and standard, mainly based on the R-CHOP regimen that produces complete remission (CR) rates of around 70–90% [1,2], and 5-year progression-free survival (PFS) and overall survival (OS) of around 60–70% [3]. Still, this means that the standard therapy fails for 30–40% of patients in the long term, so new approaches with better treatment outcomes, for example, the use of novel drugs or other bioactive compounds, are still needed. In this context, the most critical point is to early identify patients at high risk of failure with standard therapy.

MicroRNAs (miRNAs) are non-coding RNAs with a typical length between 19 and 24 nucleotides, which usually regulate the expression of genes by inactivating their corresponding messenger RNA [4]. Various studies showed a central role of certain miRNAs in the development of cancer [5], making miRNA microarrays a potential tool to identify biomarkers during early detection, diagnosis, and follow-up of cancer patients, including lymphoma patients. Specifically, in DLBCL, miRNAs involved in lymphomagenesis have been identified, although comprehending their biological function continues to be a challenge [4,6,7]. Nevertheless, the pathogenetic effect of some of these miRNAs, such as miR-125a [8], miR-17-92 cluster [9], or miR-155 [10], is well characterized. In addition, some studies suggest that certain miRNAs, specifically miR-21 and miR-155, which differentiate between germinal center and activated B cell subtypes, play a role as prognostic markers in DLBCL [11,12,13]. However, these studies included only a reduced number of patients and still need to be expanded. Other studies selected miRNAs with prognostic value on DLBCL based on differential expression of miRNAs regarding OS, cell origin, non-tumoral lymphoid tissue, the comparison of different histologies or miRNAs identified in other malignancies [14,15,16,17,18,19,20,21]. However, none of those experimental designs were directly focused on resistance to chemotherapy. In this context, our goal was to analyze which miRNAs might be associated with chemosensitivity or chemoresistance to first line treatment in DLBCL patients, including their correlation with standard prognostic factors at diagnosis and their role in survival as well as to corroborate their biological activity in vitro in a DLBCL cell line.

## 2. Materials and Methods

### 2.1. Patients and Samples

Patients (*N* = 156) homogeneously treated with R-CHOP with or without radiotherapy in three Spanish centers were included in the study. In order to avoid selection bias tissue samples were retrospectively obtained from Pathology Department registry and only those patients with valid genomic material in formalin-fixed-paraffin-embedded tissue and with available clinical data were considered. Patients who had received other chemotherapy schemes and patients with severe concomitant diseases or CNS infiltration were excluded.

### 2.2. Study Design

After obtaining the samples, RNA purification from formalin-fixed paraffin-embedded tissue sections was performed with RecoverAll^TM^ Total Nucleic Acid Isolation Kit (ThermoFisher Scientific, Madrid, Spain). Later, miRNA screening was performed using GeneChip™ miRNA 4.0 microarrays (ThermoFisher Scientific, Madrid, Spain), which included 6631 miRNA-related probes (2578 mature miRNAs, 2025 pre-miRNAs, 1996 Human snoRNA, CDBox RNA, and other non-coding RNAs).

Subsequently, bioinformatic analysis of the miRNAs with differential expression was conducted in a discovery group including two cohorts (patients with durable complete remission (CR) versus refractory or early relapsing (RR) patients). Differentially expression miRNAs were validated in the entire series using quantitative RT-PCR with Sybr Green probes (EPIK miRNA Select Lo-ROX kit). The relevance of these miRNAs as predictors of EFS and OS as well as their relationship with standard prognostic factors in DLBCL were also determined.

### 2.3. Cell Culture

To evaluate the biological activity of the selected miRNAs, we performed in vitro experiments with DLBCL cell lines. The Human DLBCL cell line U-2932 (ACC 633) was obtained from the DSMZ (Leibniz-Institut DSMZ German Collection of Microorganisms and Cell Cultures GmbH, Braunschweig, Germany). U-2932 cells, assigned to ABC-like lymphoma subtype (activated B-cell), have been described in the literature to overexpress BCL2, BCL6, and p53, which may play a role in tumorigenesis and drug resistance [22]. DLBCL cells were maintained at 0.5–1.5 × 10^6^ cells/mL in RPMI-1640 medium with 2 mM l-glutamine (BIOWEST, LabClinics S.A., Barcelona, Spain) supplemented with 10% (*v*/*v*) heat inactivated fetal bovine serum (BIOWEST), 100 units/mL penicillin and 100 µg/mL streptomycin (BIOWEST) and 10 mM HEPES (Sigma-Aldrich, Madrid, Spain) at 37 °C in 5% CO_2_. Controls regarding contamination with *Mycoplasma* were carried out routinely.

### 2.4. Transfection with miRNAs

DLBCL cells were reverse transfected separately in complete medium with 100 nM of mirVana miRNA mimics or inhibitors (hsa-miR-1244, hsa-miR-193b-5p and hsa-miR-1231), miRNA negative control #1 or miRNA positive control (mirVana miRNA mimic miR-1 positive control) (Thermo Fisher Scientific, Madrid, Spain) using Viromer^®^GREEN reagent (Lipocalyx GmbH, Halle, Germany), according to the transfection protocol specified by the manufacturer. Cells were immediately placed in an incubator at 37 °C in 5% CO_2_ and used for further experiments 48 or 72 h after transfection. Fluorescence (fluorescein)-labeled oligonucleotide, BLOCK-iTTM Alexa Fluor^®^ Red Fluorescent Oligo (Invitrogen, Madrid, Spain), was used for monitoring cellular transfection efficiency. Images were taken at 40-fold magnification (Epifluorescence Microscope Nikon Eclipse TE 2000-S) and the percentage of fluorescent cells was calculated from four different fields from each well. The transfection efficiency was around 90% (Figure S1).

### 2.5. Cell Viability

To evaluate the effect of miRNA mimics or inhibitors on cell viability after 48 h of transfection, 6 × 10^4^ DLBCL cells in 100 µL of complete medium were reverse transfected separately in 96-well E-plates with miRNA mimics, miRNA inhibitors, or miRNA mimic negative control #1 (miR control (−)), as previously described.

In vitro treatment was performed similar to previously published studies regarding compound ratios and CHOP concentration [11,23]. The final concentrations of cyclophosphamide, doxorubicin (hydroxydaunorubicin), and vincristine were in the range of mean 24 h plasma concentrations reported in patients [24], albeit somewhat higher or lower depending on the specific compound. Twenty-four hours after transfection, cells were treated with vehicle (1% DMSO) or with increasing concentrations of CHOP (range 0.3–10 µg/mL) (cyclophosphamide, hydroxydaunorubicin/adriamycin, vincristine sulfate, and prednisone) (Sigma-Aldrich, Madrid, Spain) for 48 h. The ratio of the four drugs was 80 mg/5.33 mg/0.16 mg/5.77 mg) and was determined based on the ratio used in clinical application. The CHOP concentration resulting in 50% inhibition of cell viability (IC_50_) was determined using GraphPad software.

At the end of transfection and/or CHOP treatment, the methylthiazoletetrazolium (MTT) method was applied to assess cell viability using the CellTiter 96^®^AQueous One Solution (Promega, Madison, WI, USA) following the manufacturer’s protocol. Absorbance at 490 nm was measured using a multi-well scanning spectrophotometer (PowerWave HT, Bio-tek, Winooski, VT, USA). The mean percentage of cell viability relative to that of non-transfected or cells transfected with miR control (−) in the absence or presence of 1% DMSO (vehicle-treated cells) was projected from data of at least three independent experiments performed in triplicate.

### 2.6. Total RNA Extraction

For total RNA extraction, 3.2 × 10^5^ U-2932 cells in 500 µL of complete medium were reverse transfected in 24-well E-plates with miRNA mimics or inhibitors, and miRNA negative control #1 or miRNA mimic miR-1 positive control, respectively, for 48 h, as described. Total RNA was isolated using the RNeasy Mini Kit (Qiagen, Hilden, Germany) according to the manufacturer’s instructions. RNA purity and concentration were evaluated spectrophotometrically using NanoDrop ND-2000 (ThermoFisher Scientifics; Waltham, MA, USA). The absorbance 260/280 ratio was greater than 2.0 in all cases, and the transfection efficiency was 92 ± 0.97%.

### 2.7. Quantitative Real-Time PCR

RNA (30 ng/µL; 120 ng total) was reverse-transcribed into cDNA using High-Capacity cDNA Reverse Transcription Kit (Applied Biosystems). Subsequent quantitative real-time PCR reactions were performed using the *PTK9* primers (Fw 5′-GATTCCTTTGTTTTACCCCTGTTGGAG-3′ and Rv 5′-GTCAAAAAAATGTTGTAT GCAGCAA-3′) (TIB MOLBIOL, Berlin, Germany) (CFX96 Real-Time System, C1000 Thermal Cycler, BIO-RAD). Relative gene expression was normalized to β-glucuronidase (GUS) (Fw 5′-GAAAATATGTGGTTGGAGA-3′ and Rv 5′-CCGAGTG AAGATCCCCTTTTTA-3′).

### 2.8. Screening of Differentially Expressed Genes (DEG) and Gene Set Enrichment Analysis (GSEA)

Gene expression profiling (GEP) was performed in patients using GeneChip™ HG U133 Plus 2.0, and in cell lines using Clariom™ S. Array normalization was carried out using Transcriptome Analysis Console (TAC) Software from ThermoFisher and oligo package from Bioconductor. Gene set enrichment analysis with Gene Ontology terms was performed according to the expression profile between CR and RR patients using clusterProfiler package from Bioconductor. Only those pathways with genes for which a consistent up- or downregulation was detected in both patients and U-2932 cells were considered.

### 2.9. Statistical Analysis

Qualitative variables were expressed as frequencies and percentages. Comparisons between qualitative variables were performed using the Fisher’s exact test or the chi-squared test. Comparisons between quantitative and qualitative variables were performed through non-parametric tests (U of Mann–Whitney or Kruskal–Wallis).

Time to event variables (OS and EFS) were measured from the date of therapy onset and were estimated according to the Kaplan–Meier method. Comparisons between the variables of interest were performed by the log-rank test. Multivariate analysis with the variables that appeared to be significant in the univariate analysis was carried out according to the Cox proportional hazard regression model. All *p*-values reported were two-sided, and statistical significance was defined at *p* < 0.05.

For the in vitro studies, the results were expressed as the mean ± S.E.M. (standard error of the mean). Student’s two-tailed *t*-test for unpaired data or One-Way ANOVA, followed by Newman–Keuls multiple comparison test were used for statistical evaluations. Differences were considered statistically significant at *p* < 0.05.

## 3. Results

### 3.1. Patients’ Characteristics

Diagnostic and clinical data of 156 patients homogeneously treated with R-CHOP with or without radiotherapy were retrospectively selected from three Spanish Centers (Son Espases University Hospital (HUSE), *n* = 113; Son Llàtzer Hospital (HSLL), *n* = 25; and Hospital del Mar (HM), *n* = 18). Patients’ main characteristics of the global group and both discovery and validation cohorts are detailed in Table 1. In summary, median age was 60 years (range 15–87), 56% of the patients had advance stage (III–IV), 36% bulky mass, and 32% high revised International Prognostic Index (R-IPI).

### 3.2. Identification of miRNAs with Prognostic Role

A total of 96 formalin-fixed paraffin-embedded samples suitable for sections and RNA extraction were obtained (69 samples from HUSE, 19 from HSLL, and 8 from HM). To determine those miRNAs with prognostic relevance, a discovery group was used including 12 patients with poor prognosis (high tumor burden and advanced stage (III–V and R-IPI > 2)). From these 12 patients with poor prognosis, six were chemoresistant (refractory or early relapse < 12 months (RR)) and six patients were chemosensitive (durable CR > 3 years). Clinical characteristics of the discovery cohort for the identification of miRNAs with differential expression in chemo-resistance are summarized in Table 2. Overall response rate was 100% in the CR subgroup and 83% in the RR subgroup and relapse rate 0% in the CR subgroup and 100% in the RR subgroup.

### 3.3. miRNA Microarray

**Figure 1:**
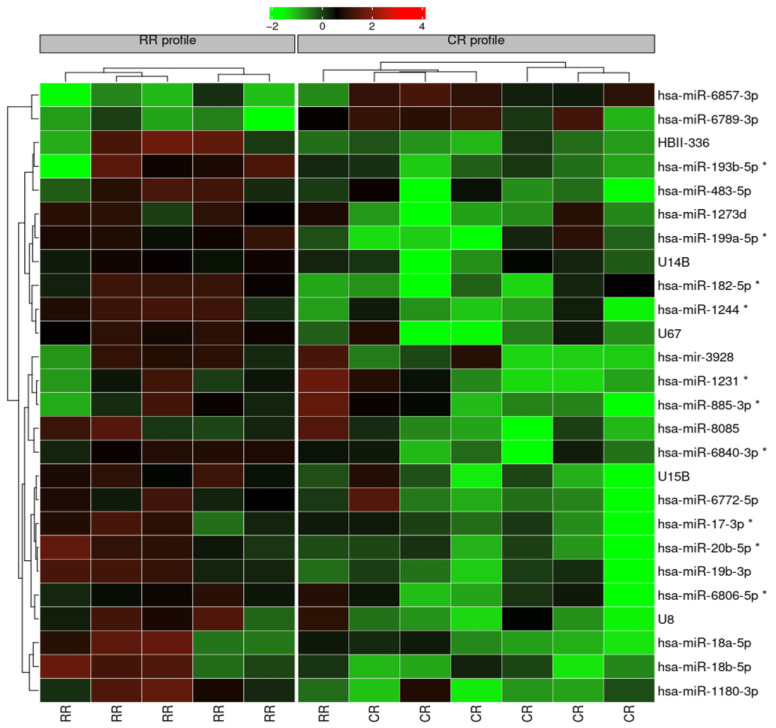
Heatmap of unsupervised hierarchical clustering to visualize the miRNAs expression profiles. Selected miRNAs that were used for further analysis in the validation cohort are highlighted with an asterisk (*).

By the use of microarray assays including 6631 miRNA-related probes, those miRNAs with the statistically most significant differences in expression (fold-change ≥ 2.0, *p* < 0.01) were defined as initial set of prognostic miRNA candidates. These 26 deregulated miRNAs were then analyzed via unsupervised hierarchical clustering to visualize the expression profile via heatmap (Figure 1).

Through a selection of miRNAs based in the first place on their relationship to relevant pathways for drug resistance in lymphoma, but also taking into account their fold change, a final list of 10 miRNAs that were overexpressed in the RR subgroup was obtained (Table 3). These differentially expressed miRNAs were then validated in the entire series using quantitative RT-PCR with the aim to evaluate their prognosis role in a large, homogenously treated cohort. The validation cohort finally consisted of 68 samples with adequate material for RT-PCR.

### 3.4. Validation of miRNAs in the Validation Cohort and Their Prognostic Role on Survival

Survival univariate analysis was performed including standard clinical prognostic factors as well as the 10 selected miRNAs (Table 4). Event-free survival (EFS) was influenced by seven of these miRNAs (miR-20b-5p, miR-1244, miR-6840-3p, miR-1231, miR-193b-5p, miR-6860-5p, and miR-199a-5p) and OS was influenced by six of them (miR-1244, miR-1231, miR-193b-5p, miR-885-3p, miR-182-5p and miR-199a-5p). miR-20b-5p, belonging to cluster miR-17-92, was differentially expressed in the RR subgroup and showed a significant impact on EFS in our series. From 10 tested miRNAs, only three had a significant prognostic role on PFS, EFS, and OS (miR-1244, miR-1231 and miR-193b-5p).

**Figure 2:**
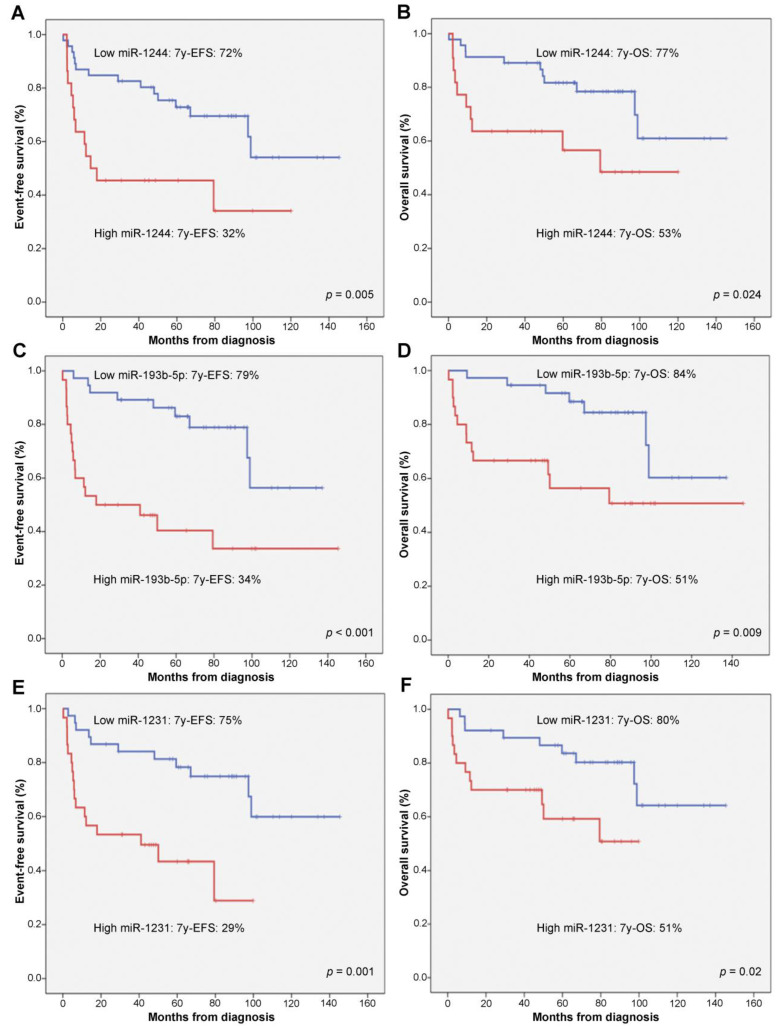
High levels of miR-1244, miR-193b-5p, or miR-1231 were related to worse event-free survival (EFS) and overall survival (OS). The expression of miR-1244 (**A**,**B**), miR-93b-5p (**C**,**D**), and miR-1231 (**E**,**F**) was studied for their impact on EFS and OS, respectively, in our series of patients with diffuse large B-cell lymphoma (DLBCL). EFS and OS were compared using the Kaplan–Meier curves and log rank test between the high (red line) and low (blue line) expression groups determined by each miRNA-specific threshold. *p* value indicates statistical significance.

miR-1244 was the most overexpressed of these three miRNAs (Fold change: 6.74). In our series, and considering clinical variables, overexpression of this miRNA was associated with advanced stage (III–IV) (*p* = 0.01), B symptoms (*p* = 0.005), and poorer TS (*p* = 0.026). On the other hand, the data confirmed that overexpression of miR-193b-5p had a clinical association with higher LDH (*p* = 0.003), B2M (*p* = 0.004), ESR (*p* = 0.041), B symptoms (*p* = 0.019), IPI (*p* = 0.028), TS (*p* < 0.001), and ECOG PS > 1 (*p* = 0.009). In addition, miR-1231 had an independent prognostic role not related to any clinical variable or the other miRNAs in our series. The overexpression of all these three miRNAs (miR-1244, miR-193b-5p, and miR-1231) carried worse EFS and OS (Figure 2).

Multivariate analysis was performed including clinical and biological variables (Table 5) and it could be observed that overexpression of miR-1231, high R-IPI, and reduced relatively dose intensity (RDI) > 15% were independently associated with worse EFS and OS.

### 3.5. Biological Activity of miR-1244, miR-193b-5p, and miR-1231 on DLBCL Cells In Vitro

To evaluate the biological activity of miR-1244, miR-193b-5p, and miR-1231, which had a prognostic role for PFS, EFS, and OS in homogeneously treated DLBCL patients, the effect of these miRNAs on DLBCL cell viability was for the first time studied in cell culture. Reverse transfection of U-2932 cells with BLOCK-iT^TM^ Alexa Fluor^®^ Red Fluorescent Oligo and Viromer Green reagent achieved a high percentage of 89.6 ± 0.7% of transfected cells after 48 h (Figure S1). Moreover, transfection of U-2932 cells with the miRNA mimic miR-1 positive control was able to decrease the expression of its target gene *PTK9* (tyrosine kinase 9) (Figure S1), demonstrating that the optimized transfection conditions allow for miRNA functional studies.

**Figure 3:**
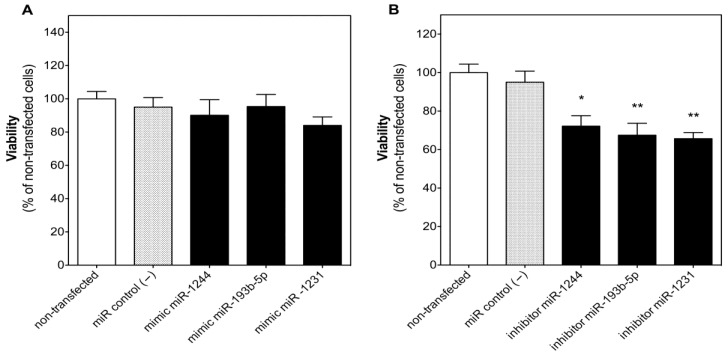
Inhibition, but not expression, of miR-1244, miR-193b-5p or miR-1231 reduced cell viability in human diffuse large B-cell lymphoma (DLBCL) cells. U-2932 cells were reverse transfected separately with 100 nM of miR-1244, miR-193b-5p, or miR-1231 miRNA mimics (**A**) or inhibitors (**B**) for 48 h. In parallel, another set of cells were reverse transfected with miRNA negative control #1 (miR control (−)) or left entirely untreated. Cell viability was determined as detailed in Materials and Methods. Each column represents mean ± SEM of 3 independent experiments performed in triplicate, and normalized to cells transfected with miR control (−) (taken as 100%). * *p* < 0.05 and ** *p* < 0.001 versus cells transfected with miR control (−).

The miRNA negative control (100 nM) (miR control (−)) did not change the viability of U-2932 cells with respect to non-transfected (NT) cells after 48 h of transfection, discarding an adverse effect of the transfection method itself on cell viability (Figure 3). Transfection of miR-1244, miR-193b-5p or miR-1231 (100 nM) mimics did not significantly change the viability of U-2932 (Figure 3A) with respect to miR control (−)-transfected cells. However, the inhibition of these three miRNAs of endogenous origin with their respective inhibitors significantly decreased U-2932 cell viability after 48 h by 24.1 ± 5.7%, 28.9 ± 6.5%, and 30.9 ± 3.3%, respectively (Figure 3B), supporting the idea that these miRNAs are involved in the survival of these tumor cells.

**Figure 4:**
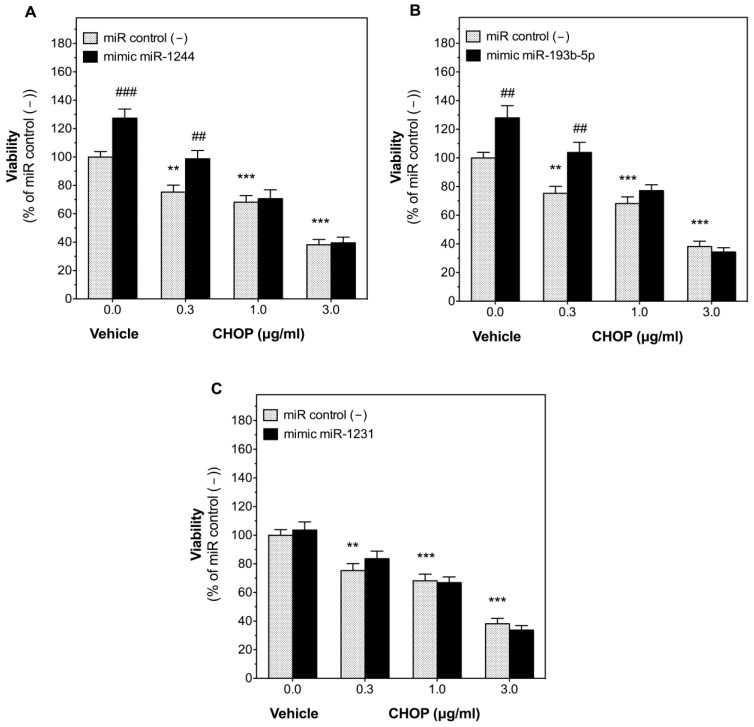
Expression of miR-1244 and miR-193b-5p, but not miR-1231, blocked the antitumoral effect of CHOP in human diffuse large B-cell lymphoma (DLBCL) cells. U-2932 cells were reverse transfected separately with 100 nM of miR-1244 (**A**), miR-193b-5p (**B**), or miR-1231 (**C**) mimics or with miRNA negative control #1 (miR control (−)). 24 h after transfection, cells were treated with vehicle (1% DMSO) or with increasing concentrations (0.3, 1, and 3 µg/mL) of CHOP. Cell viability was determined as detailed in Materials and Methods. Each column represents mean ± SEM of 4 independent experiments performed in triplicate, and normalized to cells transfected with miR control (−) (taken as 100%). ** *p* < 0.01 and *** *p* < 0.001 versus cells transfected with miR control (−) and treated with vehicle. ## *p* < 0.01 and ### *p* < 0.001 versus their respective cells transfected with miR control (−).

The effect of miR-1244, miR-193b-5p, or miR-1231 on the response of DLBCL cells to CHOP treatment was then evaluated. Transfection of U-2932 with miR control (−) did not change cell sensitivity to CHOP that reduced viability dose-dependently with an IC_50_ of 1.45 μg/mL in non-transfected and miR control (−)-transfected cells (Figure S2). Transfection of U-2932 cells with miR-1244 or miR-193b-5p mimics increased cell viability in the presence of vehicle (1% DMSO) by 27.4 ± 6.4% and 27.9 ± 8.4% (Figure 4), respectively. Even more importantly, these miRNAs completely blocked the reduction of cell viability by CHOP (0.3 μg/mL) after 48 h of treatment. On the contrary, miR-1231 mimic did not significantly change the inhibitory effect of CHOP on U-2932 cell viability at any of the concentrations studied (0.3, 1, and 3 μg/mL).

### 3.6. Screening of Differentially Expressed Genes (DEG) and Gene Set Enrichment Analysis (GSEA)

**Figure 5:**
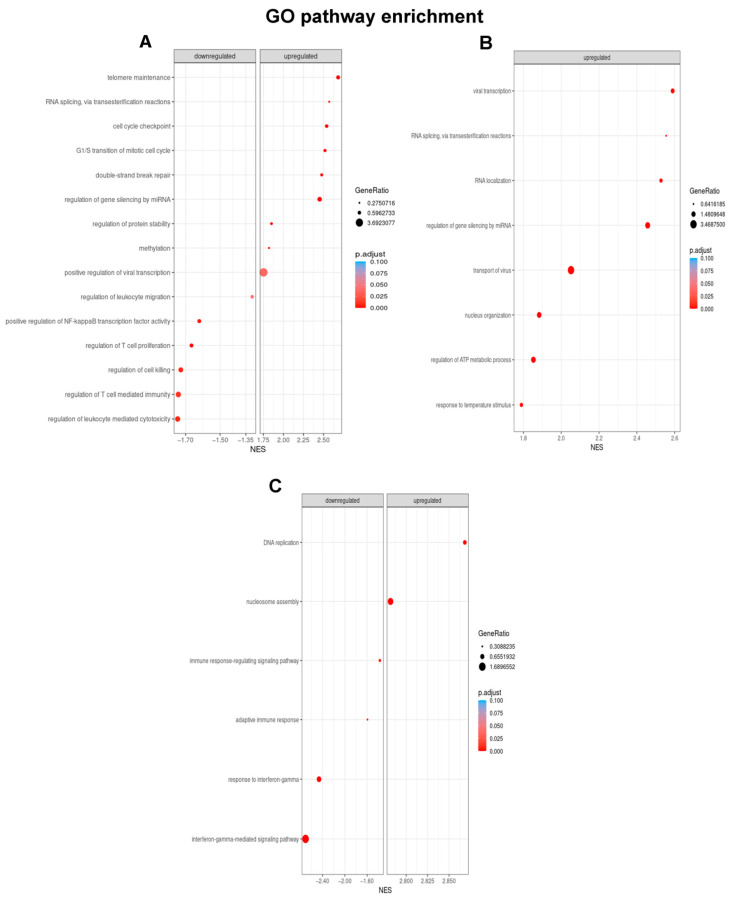
Gene set enrichment analysis with Gene Ontology terms was performed according to the expression profile between CR and RR patients using clusterProfiler package from Bioconductor. Only those pathways with genes for which a consistent up- or downregulation was detected in both patients and U-2932 cells were considered. Gene Ontology categories altered by miR-1244 (**A**), miR-193-5p (**B**), and miR-1231 (**C**) including their corresponding NES (Normalized Enrichment Score) values are indicated in the respective panels. The higher the enrichment score the stronger is the displacement of genes belonging to certain GO categories towards either end of the ranked list with positive values representing up- and negative values downregulation.

When considering in parallel the results obtained in the discovery group of patients as well as in miRNA-transfected U-2932 cells, GSEA revealed that the studied miRNAs were related to pathways with clinical relevance of the study objective. An overview of selected gene subsets related to lymphoma (Figure 5) gives a general idea about the nature of these alterations.

Increased levels of miR-1244 led to a robust and statistically significant upregulation of pathways related to cell cycle activation and double-strand break repair (Figure 5A). Moreover, T-cell mediated immunity and immune surveillance pathways were downregulated, which could lead to tumor evasion. Nevertheless, DEG did not reveal any drastically modified expression patterns of single genes in these pathways, although certain genes are involved in more than one pathway. For instance, miR-1244 induced upregulation of the *PRKDC* gene (Protein Kinase, DNA-activated, Catalytic subunit), which is linked to various altered pathways, although its increase in gene expression was not statistically significant.

High levels of miR-193b-5p in patients as well as in cells were clearly related to gene enrichment in eight pathways (Figure 5B), two of them also affected by miR-1244. In this case, all pathways were upregulated. Again, DEG did not point out isolated genes as key figures, but implied an incremented expression of numerous pathway genes, such as *NUP98* (Nucleoporin 98 and 96 Precursor), which promotes cell cycle progression and is linked to all enriched gene sets.

Finally, miR-1231 altered six pathways, none of which was influenced by one of the other two miRNA (Figure 5C). In this context, miR-1231 downregulated four pathways related to immune surveillance whereas two proliferative pathways were upregulated. For example, DEG data showed, that miR-1231 induced downregulation of *HLA-DRB5* (Major Histocompatibility Complex, Class II, DR Beta 5), a gene that is involved in tumor cell recognition through T lymphocytes.

## 4. Discussion

Early detection of patients with a high risk of chemotherapy failure is an indispensable first step to develop new treatment strategies. In this context, miRNAs have been considered possible biomarkers, but a recent systematic review displayed a disillusioning picture as to the lack of congruence regarding the identified miRNAs [25]. An important issue was that miRNAs can be used as biomarkers for diagnosis, classification, prognosis, or treatment outcome, which leads to different sets of biomarkers and a limited number of available studies in each category. In addition, different studies identified different miRNAs and the only consistent result was the upregulation of miR-21 as a biomarker for diagnosis. Surprisingly, the results of its significance for treatment outcome were contradictory between studies and it was argued that the latter might be related to increased expression of this miRNA in patients with DLBCL stage I/II compared to stage III/IV leading to an observational bias. In general, this means that miRNA expression might not always be stable during tumor progression possibly provoking conflicting study results. In this regard, our study avoided mixing of different DLBCL stages to guarantee congruent and clearly defined results. A more recent systematic review including more studies came to similar results and concluded that there is currently insufficient evidence, in particular, with respect to prediction of treatment response [26]. In this context, most studies analyze cell-free circulating miRNAs, and only very few reports are based on tissue samples [27]. Although this is understandable because blood samples are an easy, non-invasive diagnostic procedure, the obtained results might not fully correlate with the miRNA pattern of the tumor microenvironment meaning that overexpression rates could vary depending on the sampling method and, thus, affect conclusions [28].

The selection of miRNAs in our work was straightforwardly focused on drug resistance to first line treatment and followed a novel systematic approach using tissue samples. Two groups of DLBCL patients, who had a similar poor prognosis (high tumor burden, unfavorable IPI and advanced stage), but totally differed regarding their response to R-CHOP treatment, were analyzed resulting in the identification of miR-1244, miR-193b-5p, and miR-1231 as novel biomarkers for the refractory/relapse group. Although the applied systematic approach is promising, the total number of analyzed patients was limited, so that these biomarkers still need to be confirmed by larger studies.

The effect of each of these three miRNAs was validated in cell culture, although the development of an efficient transfection protocol to perform functional studies with miRNA mimics or inhibitors was especially complex in DLBCL cell lines. In fact, no adequate methods have been described in the literature and low binding of the transfection complex with the cell membrane seems to hamper transfection of cells in suspension [29]. However, we were able to optimize a transfection protocol achieving high transfection efficacy without affecting cell viability or sensitivity to CHOP treatment, thus, allowing for miRNA functional studies in DLBCL suspension cells. As expected, due to our clinical data, inhibition of endogenous miR-1244, miR-193b-5p or miR-1231 with their respective inhibitors suppressed cell viability in U-2932 cells, suggesting a role of these miRNAs in the survival of DLBCL cells. More importantly, transfection of miR-1244 or miR-193b-5p mimics not only increased basal cell viability in U-2932 cells in the presence of vehicle, but also completely blocked the antitumoral effect of CHOP (0.3 μg/mL) after 48 h (Figure 4), corroborating the idea that the expression of miR-1244 or miR-193b-5p could play an important role in the cellular resistance to CHOP. Increased cell viability can be considered a form of chemoresistance as it actually counteracts cytotoxicity. However, at higher CHOP concentrations this protection most probably cannot keep up with the accumulating cytotoxic effects, which is why cell viability collapses, and the protective effect of these miRNAs is not detectable at elevated concentrations (1 and 3 μg/mL). In this context, it should be taken into account that cytotoxic experiments in cell culture are only indicative and cannot replace clinical results. For example, DMSO is a compound known to increase transport of substances across membranes [30] and the necessity to use this vehicle to dissolve CHOP most probably increased the cellular uptake of our miRNA mimics, which led to a variation of cell viability between control cells of Figure 3A and Figure 4A, respectively.

Of the three selected miRNAs, miR-1244 showed the highest fold change in our clinical series. In the literature, this miRNA has been described as differentially expressed in Burkitt lymphoma compared to non-Burkitt lymphoma (DLBCL and follicular lymphoma) and, in this context, it has been proposed as a novel molecular target in lymphomagenesis [31]. Moreover, integration of experimentally validated high-throughput data and computational predictions suggest that miR-1244 might be a part of the regulatory network of Myc, thus, linking it to genetic control of cell proliferation, apoptosis, and cell cycle [32]. In fact, in lung cancer cells, miR-1244 contributed to both the progression of cancer [33] and the development of cisplatin resistance [34].

An adverse prognostic role of miR-193b has been confirmed in head and neck squamous cell carcinoma (HNSCC) patients, since high levels of this miRNA in HNSCC tumors were associated with lower disease-free survival [35]. Interestingly, in FaDu cancer cells (HNSCC) in vitro knockdown of miR-193b substantially reduced cell proliferation and migration, as well as tumor formation in an animal model [35]. In contrast, in human ovarian cancer cells it has been suggested that miR-193b could act as tumor suppressor by inhibiting cell proliferation and inducing apoptosis [36]. The lesson to learn from such apparently contradictory observations is that the specific functional role of miRNAs (oncogene or tumor suppressor gene) may vary depending on the tumoral tissue type. Regarding hematological malignancies, increased expression of the miRNA cluster miR-193b-365 has been reported in multiple myeloma [37]. On the molecular level, some investigators have suggested that miR-193a-3p silencing, due to DNA hypermethylation by the AML1/ETO co-repressor complex, would increase the oncogenic activity of the fusion protein AML1/ETO expressed in hematopoietic cells, thereby contributing to leukemogenesis in patients with acute myeloid leukemia [38]. Our own results indicate that high miR-193b-5p levels are associated with failure of standard chemotherapy in DLBCL, but it should be noted that a very recent congress abstract linked downregulation of miR-193b-5p in pre-treatment tissue samples to refractory/relapsed DLBC [39].

Finally, miR-1231 was the third selected miRNA and is the only one with an independent prognostic value of clinical variables in multivariate analysis compared to the other miRNAs. Although some studies have linked miR-1231 to viral infections such as hepatitis B [40], others have demonstrated its oncogenic role in pancreas cancer [41], glioma [42], and prostate cancer, where its expression has been considered a prognostic factor for patients [43]. In this context, a recent study in diabetic patients with non-small cell lung cancer found that high tumor tissue levels of miR-1231 correlated to lower mortality. This is an interesting finding as diabetes mellitus is generally associated with poor OS in lung cancer patients [44]. While these results might seem contradictory to ours at first glance, it is important to bear in mind that the study did not reveal whether miR-1231 levels were linked to blood glucose levels in those patients, making it difficult to conclude how miR-1231 levels might be influenced by hyperglycemia. In this context, it is remarkable that overexpression or silencing of miR-1231 in cell culture had no statistically significant effect on cell viability of CHOP-treated U-2932 cells. Therefore, this miRNA does not seem to be directly involved in molecular mechanisms of drug resistance, but is probably overexpressed because of other cellular events in DLBCL patients being resistant to R-CHOP in our series.

miRNAs have a balancing regulatory effect on cellular processes and are therefore more likely modulating entire pathways to exert their effects than only single centerpiece genes. In line with this idea, our GSEA delivered statistically more robust data than screening for individual, highly differentially expressed genes. Contrasting our clinical with our in vitro data produced a coherent picture of affected pathways, most of them directly associated with events related to cell division and immune response, thus, reaffirming the importance that these miRNAs probably have in DLBCL patients. In fact, miR-1244 was able to change more pathways than the other two, and also showed the highest fold-change among the three miRNAs, for which reason it might play a more prominent role.

Interestingly, miR-1244 and miR-193b-5p, but not miR-1231, had an up-regulation of the RNA splicing pathway in common. It has been reported that this pathway is implicated in the cellular response to chemotherapy, which would be in line with the observed effect on resistance to R-CHOP treatment. Interestingly, these two miRNAs also showed a high NES value (>2.4) regarding regulation of gene silencing by miRNAs.

When pooling the effects it becomes clear that all downregulated pathways were associated with immune surveillance implying a possible role of the respective miRNAs in immune escape. Specially, miR-1231 seems to be mainly implicated in adaptive immune response and interferon-gamma-related pathways. Bearing in mind that miR-1231 had no effect on CHOP treatment outcome in cell culture, this could suggest an indirect role of this miRNA possibly being associated with tumor escape mechanisms and to a lesser extent proliferative cellular events. This idea is strengthened by the observed downregulation of the MHC-II isotype *HLA-DRB5* gene, which seems to have a pathogenic effect and leads to worse prognosis in a range of human tumors [45]. In fact, a recently published clinical trial found that mutational alterations and expression signatures of inflammatory pathways in R-CHOP treated non-GCB DLBCL patients were associated with proliferative signaling and poor treatment outcome. When the immunomodulatory agent lenalidomide was administered in parallel to the standard therapy (R2CHOP) the treatment outcome improved, thus, showing that chemoresistance in DLBCL patients might indeed be linked to an inflammatory environment [46].

## 5. Conclusions

In summary, we identified for the first time three novel miRNAs (miR-1244, miR-193b-5p, and miR-1231) with prognostic value on EFS and OS in patients with DLBCL, and confirmed in vitro that the downregulation of the first two inhibits cell viability and increases resistance to CHOP treatment in DLBCL cells. Only future corroboration of these biomarkers in clinical practice will show, if the discovery strategy followed in this study may generally be useful to identify further prognostic miRNAs in cancers with similar therapeutic characteristics.

## Figures and Tables

**Table 1:** Main characteristics of patients.

Variable	Global Group (*n* = 156)	Discovery Group (*n* = 12)	Validation Group (*n* = 68)
Median age (range)	60 (15–87)	46 (33–78)	54 (15–87)
Sex (M/F)	85 (55%)/71 (45%)	5 (42%)/7 (58%)	39 (57%)/29 (43%)
ECOG PS ^1^ > 1	34 (22%)	7 (58%)	16 (23%)
AA ^2^ III–IV stage	88 (56%)	11 (92%)	37 (54%)
B symptoms	51 (33%)	9 (75%)	21 (31%)
High LDH ^3^	74 (47%)	9 (75%)	34 (51%)
>1 extranodal sites	27 (17%)	3 (25%)	10 (15%)
Bulky mass	57 (36%)	3 (25%)	20 (29%)
aIPI > 1	62 (40%)	11 (92%)	26 (39%)
High R-IPI	50 (32%)	9 (75%)	20 (30%)
NCCN-IPI			
Low	26 (16%)	1 (8%)	13 (20%)
Low–intermediate	62 (39%)	2 (17%)	28 (43%)
Intermediate–high	49 (32%)	8 (67%)	21 (32%)
High	11 (7%)	1 (8%)	3 (5%)
High B2M ^4^	63 (40%)	7 (64%)	24 (37%)
Radiotherapy	54 (35%)	1 (8%)	22 (32%)

^1^ Eastern Cooperative Oncology Group Performance Status; ^2^ Ann Arbor; ^3^ Lactate dehydrogenase; ^4^ ß-2 microglobulin.

**Table 2:** Clinical characteristics and therapy related outcomes of the discovery cohort.

Variable	Refractory/Early Relapse (RR Subgroup)	Complete Remission (CR Subgroup)
Group Size	6	6
Median age (range)	50 (38–78)	44 (30–72)
Sex (M/F)	3/3	4/2
ECOG PS > 1	5 (83%)	3 (50%)
AA III–IV stage	6 (100%)	6 (100%)
B symptoms	5 (83%)	5 (83%)
High LDH	6 (100%)	5 (83%)
>1 extranodal sites	3 (50%)	1 (17%)
Bulky mass	2 (33%)	2 (33%)
a-IPI > 1	6 (100%)	6 (100%)
High B2M	3 (50%)	5 (83%)
Radiotherapy	2 (33%)	0 (0%)
5-Year-OS	0%	100%
Median PFS (95%CI)	7.6 (2.1–11.4)	NA

**Table 3:** Selected miRNAs for analysis in the overall series.

miRNA	Fold Change
hsa-miR-17-3p	3.20
has-miR-20b-5p	4.60
hsa-miR-1244	6.74
hsa-miR-6840-3p	3.09
hsa-miR-1231	2.80
hsa-miR-193b-5p	3.08
hsa-miR-6806-5p	2.17
hsa-miR-885-3p	2.27
hsa-miR-182-5p	4.74
hsa-miR-199a-5p	4.88

**Table 4:** Univariate analysis including clinical and biological variables of the validation cohort.

Variable	Range	7y-OS (95%CI)	*p*	7y-EFS (95%CI)	*p*
Age	0–60 >60	74% (58–91) 61% (43–79)	0.1	64% (46–81) 51% (33–70)	0.14
Sex	Male Female	78% (65–92) 56% (36–77)	0.25	63% (47–78) 53% (33–74)	0.76
ECOG PS	0–1 2–4	75% (62–88) 48% (23–74)	**0.037**	66% (52–80) 34% (9–59)	**0.027**
AA stage	I–II III–IV	83% (69–97) 58% (40–75)	0.067	74% (58–89) 46% (28–64)	0.08
B symptoms	No Yes	79% (66–91) 47% (23–71)	**0.019**	68% (53–82) 37% (13–61)	**0.026**
LDH	Normal High	75% (57–94) 60% (42–77)	0.072	69% (49–88) 48% (31–66)	**0.03**
Extranodal sites	0–1 >1	71% (57–84) 58% (27–90)	0.61	58% (44–72) 58% (27–90)	0.79
Bulky mass	No Yes	67% (52–81) 73% (53–94)	0.78	58% (42–74) 58% (36–81)	0.85
B2M	Normal High	78% (64–92) 58% (35–80)	0.072	67% (51–83) 51% (29–73)	0.097
R-IPI	Low Intermediate High	100% 72% (55–88) 49% (27–72)	**0.01**	100% 62% (45–80) 39% (17–61)	**0.008**
TS ^1^	0–2 3–5	82% (69–94) 44% (21–66)	**0.005**	72% (57–86) 37% (16–59)	**0.007**
Radiotherapy	Yes No	95% (87–100) 55% (39–71)	**0.024**	86% (71–100) 45% (28–61)	**0.02**
RDI ^2^	100–85% <85%	75% (62–88) 48% (20–76)	**0.002**	67% (52–81) 33% (9–59)	**<0.001**
miR-1244	0–0.31 >0.31	77% (64–90) 53% (30–76)	**0.024**	72% (58–87) 32% (9–56)	**0.005**
miR-193b-5p	0–0.27 >0.27	84% (72–97) 51% (30–71)	**0.009**	79% (64–93) 34% (14–53)	**<0.001**
miR-1231	0.215 >0.215	80% (67–93) 51% (28–73)	**0.02**	75% (60–89) 29% (2–55)	**0.001**
miR-20b-5p	0–0.6 >0.6	78% (61–95) 63% (46–79)	0.21	73% (54–92) 49% (32–65)	**0.046**
miR-17-3p	0–0.06 >0.06	57% (20–94) 70% (57–83)	0.35	21% (0–56) 62% (48–76)	0.053
miR-182-5p	0–0.065 >0.065	92% (78–100) 61% (47–76)	**0.043**	52% (34–70) 62% (42–82)	0.12
miR-199a-5	0.7 >0.7	81% (67–95) 55% (36–74)	**0.035**	69% (53–86) 45% (26–64)	**0.04**
miR-6840-3p	0–0.41 >0.41	69% (55–84) 67% (45–89)	0.72	68% (53–82) 33% (9–57)	**0.01**
miR-885-3p	0–0.285 >0.285	79% (65–94) 52% (32–73)	**0.008**	70% (45–95) 52% (36–67)	0.12
miR-6806-5p	0–0.245 >0.245	72% (56–87) 65% (45–84)	0.43	69% (54–85) 42% (20–63)	**0.019**

^1^ Tumor Score; ^2^ Relative Dose Intensity; statistically significant results (*p* < 0.05) are highlighted in bold, *n* = 68.

**Table 5:** Multivariate analysis including clinical and biological variables.

Variable	EFS HR (95%CI)	*p*
High R-IPI	3.8 (1.7–8.5)	0.001
Reduced RDI > 15%	4.3 (1.9–9.6)	<0.001
miRNA-1231	5.6 (2.3–13.6)	<0.001
**Variable**	**OS** **HR (95%CI)**	***p***
High R-IPI	4.2 (1.7–10.4)	0.002
Reduced RDI > 15%	4.7 (1.9–11.8)	0.001
miRNA-1231	4.4 (1.6–12.2)	0.004

## Data Availability

The data presented in this study are available on request from the corresponding author. The data are not publicly available due to privacy issues.

## Supplementary Materials

The following supporting information can be downloaded at: https://www.mdpi.com/article/10.3390/cancers14041065/s1, Figure S1: Transfection efficiency and functional study with the miRNA mimic miR-1 positive control; Figure S2: Transfection of human diffuse large B-cell lymphoma (DLBCL) cells with miRNA negative control (miR control (−)) did not alter its sensitivity to CHOP.
