# Supplementary material for: Screening for Prognostic microRNAs Associated with Treatment Failure in Diffuse Large B Cell Lymphoma
Source: Cancers (Basel). 2022 Feb 20;14(4):1065. doi: 10.3390/cancers14041065 (PMC8870558; doi:10.3390/cancers14041065)
Supplement: Supplementary file 1 [file cancers-14-01065-s001.zip › cancers-1568717-supplementary.pdf]

cancers

MDPI

Article

Screening for Prognostic microRNAs Associated with Treatment Failure in Diffuse Large B Cell Lymphoma

Leyre Bento, Oliver Vögler, Adriana Sas-Barbeito, Josep Muncunill, Teresa Ros, Jordi Martínez, Adriana Quintero-Duarte, Rafael Ramos, Víctor Jose Asensio, Concepción Fernández-Rodríguez, Antonio Salar, Alfons Navarro, Raquel del Campo, Javier Ibarra, Regina Alemany and Antonio Gutiérrez

Supplementary Figures

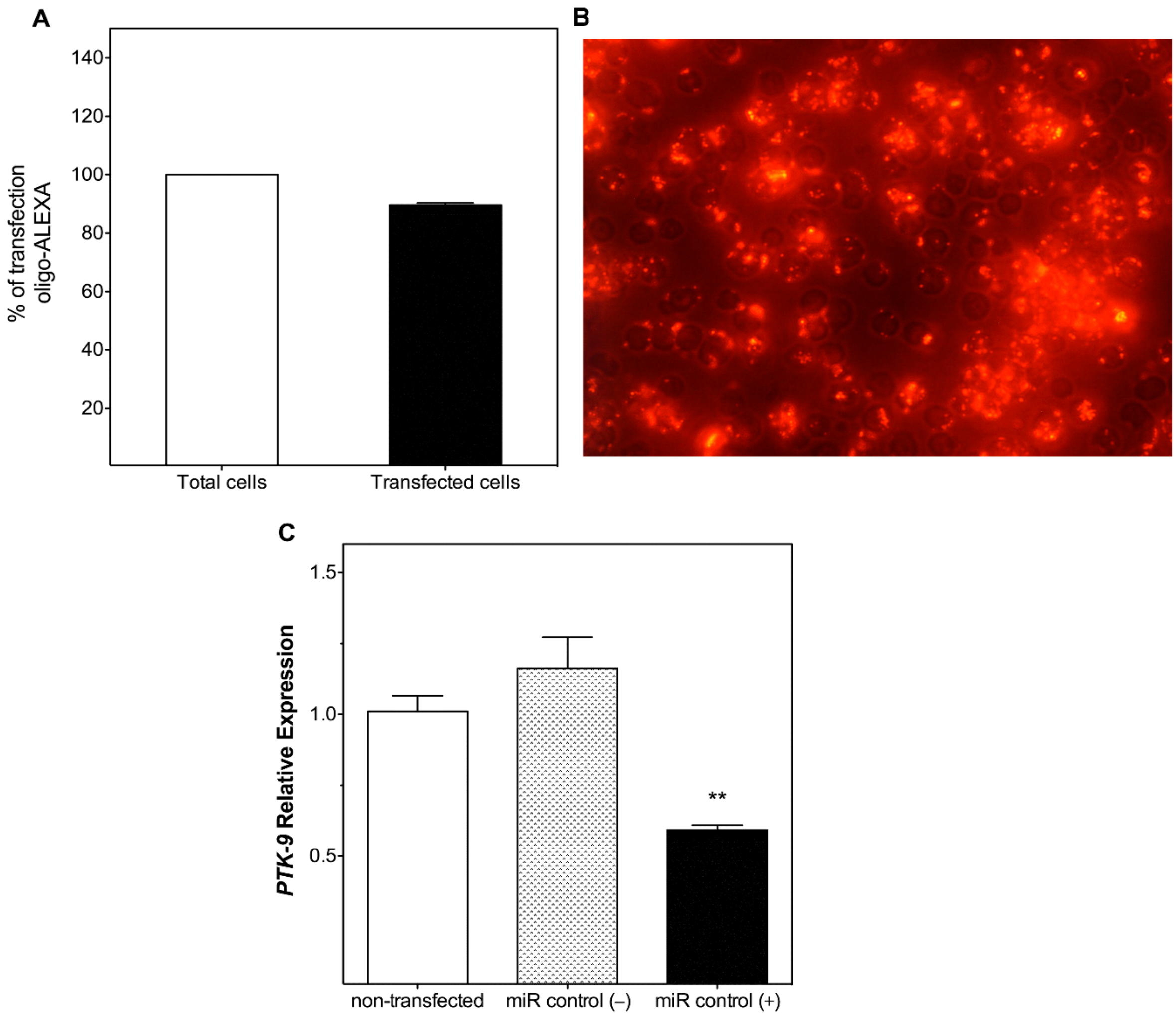

Figure S1. Transfection efficiency and functional study with the miRNA mimic miR-1 positive control. U-2932 cells were reverse transfected with the fluorescence (fluorescein)-labelled oligonucleotide, BLOCK-iTTM Alexa Fluor® Red Fluorescent Oligo (100 nM) using Viromer Green reagent as described in Materials and Methods. After a wash with PBS, images were taken at 40-fold with an Epifluorescence Microscope (Nikon Eclipse TE 2000-S) (B) and the percentage of fluorescent cells was calculated from four different fields from each well (A). For functional studies, U-2932 cells were reverse transfected with 100 nM of the miRNA mimic miR-1 positive control (miR control (+)) or with miRNA negative control (miR control (−)) for 48 h. In these experiments, after total RNA extraction, *PTK9* gene expression was quantified

using quantitative Real-Time PCR as described in Material and Methods considering mRNA levels of non-transfected cells as basal, 1-fold expression of *PKT9*. Each value represents mean ± SEM of 3 independent experiments performed in duplicate. (C) $^{**}p < 0.01$ versus both cells transfected with miR control (−) or non-transfected cells.

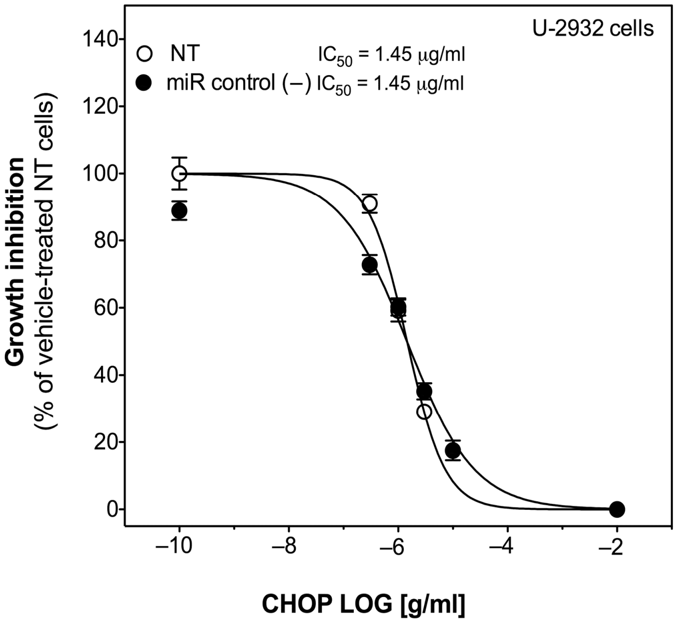

Figure S2. Transfection of human diffuse large B-cell lymphoma (DLBCL) cells with miRNA negative control (miR control (−)) did not alter its sensitivity to CHOP. U-2932 cells were reverse transfected with miR control (−) (100 nM) or left entirely untreated (Non-transfected cells) as described in Material and Methods. After 24 h of transfection, cells were treated with vehicle (1% DMSO) or with increasing concentrations of CHOP (range 0.3 - 10 µg/ml) (cyclophosphamide, hydroxydaunorubicin/adriamycin, vincristine sulfate, and prednisone) for another 48 h. The ratio of the four drugs was 80 mg/ 5.33 mg/ 0.16 mg/ 5.77 mg). Cell viability was assessed as described in Materials and methods. Each value represents mean ± SEM of 3 independent experiments performed in triplicate. The compound concentration resulting in 50% inhibition of cell viability (IC_{50}) was determined using GraphPad software.
